# On-Chip Cellomics Assay Enabling Algebraic and Geometric Understanding of Epigenetic Information in Cellular Networks of Living Systems. 1. Temporal Aspects of Epigenetic Information in Bacteria

**DOI:** 10.3390/s120607169

**Published:** 2012-05-30

**Authors:** Kenji Yasuda

**Affiliations:** Institute of Biomaterials and Bioengineering, Tokyo Medical and Dental University, 2-3-10 Kanda-Surugadai, Tokyo 101-0062, Japan; E-Mail: yasuda.bmi@tmd.ac.jp

**Keywords:** on-chip cellomics, genetic information, epigenetic information, algebraic viewpoint, geometric viewpoint, microchamber array, optical tweezers, agarose microchamber array, multi-electrode array (MEA), photo-thermal etching, *Escheirchia coli* (*E. coli*), inheritance, population effect, community effect, adaptation, variability, individuality

## Abstract

A series of studies aimed at developing methods and systems of analyzing epigenetic information in cells and in cell networks, as well as that of genetic information, was examined to expand our understanding of how living systems are determined. Because cells are minimum units reflecting epigenetic information, which is considered to map the history of a parallel-processing recurrent network of biochemical reactions, their behaviors cannot be explained by considering only conventional DNA information-processing events. The role of epigenetic information on cells, which complements their genetic information, was inferred by comparing predictions from genetic information with cell behaviour observed under conditions chosen to reveal adaptation processes, population effects and community effects. A system of analyzing epigenetic information was developed starting from the twin complementary viewpoints of cell regulation as an “algebraic” system (emphasis on temporal aspects) and as a “geometric” system (emphasis on spatial aspects). Exploiting the combination of latest microfabrication technology and measurement technologies, which we call on-chip cellomics assay, we can control and re-construct the environments and interaction of cells from “algebraic” and “geometric” viewpoints. In this review, temporal viewpoint of epigenetic information, a part of the series of single-cell-based “algebraic” and “geometric” studies of celluler systems in our research groups, are summerized and reported. The knowlege acquired from this study may lead to the use of cells that fully control practical applications like cell-based drug screening and the regeneration of organs.

## 1. Introduction

**Figure 1.**
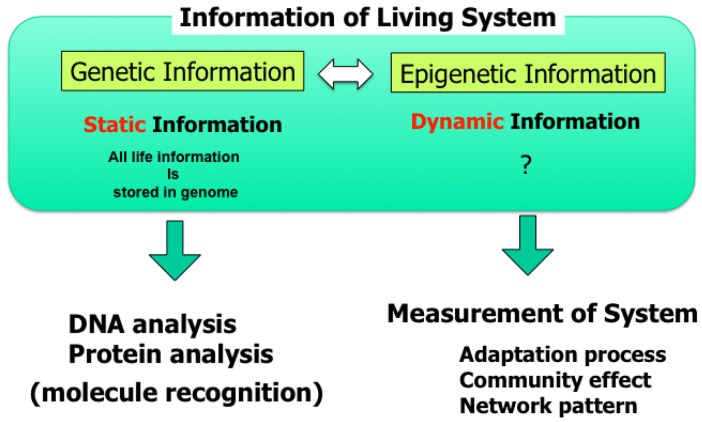
Epigenetic information: complementary to genetic information. As epigenetic information is based on the functional pathway information, it is sometimes quite difficult to measure using the conventional destructive molecule-based analysis. To measure the epigenetic information, re-constructive approach of cell network model with non-destructive long-term measurement is one of the powerful methods.

Knowledge about living organisms has increased dramatically during the 20th century and has produced the modern disciplines of genomics and proteomics. However, despite these advances, a great challenge remains in learning how the different living components of a cell are integrated and regulated. As we move into the post-genomic period, the complementarity of genomics and proteomics will become more apparent, allowing the connections between them to be exploited. However, neither genomics nor proteomics based only on genomic information can provide the knowledge needed to interconnect molecular events in living cells. The cells in a group are individual entities, and differences arise even among cells with identical genetic information that have grown under the same conditions. These cells respond differently to perturbations [1]. Why and how do these differences arise? Cells are minimum units determining their responses through genetic and epigenetic information like the history of interactions between them and fluctuations in environmental conditions affecting them. To understand the rules underlying possible differences occurring in cells, we need to develop methods of simultaneously evaluating both the genetic and epigenetic information (Figure 1).

In other words, if we are to understand adaptation processes, population effects (a group of same cells) and community effects (a group of different cells), and the meaning of cell network patterns, we need to analyze their epigenetic information. We thus started a project focusing on developing a system that could be used to evaluate the epigenetic information in cells by continuously observing specific examples and their interactions under controlled conditions. The importance of understanding epigenetic information is expected to become apparent in cell-based biological and medical fields like cell-based drug screening and the regeneration of organs from stem cells, fields where phenomena cannot be interpreted without taking epigenetic factors into account.

**Figure 2.**
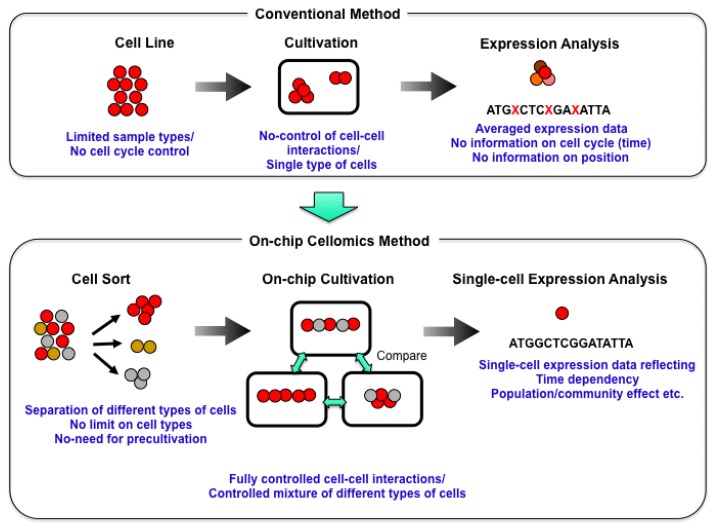
Our strategy: on-chip cellomics analysis. In the conventional cell-based studies, cell lines were usually used for acquiring the same type of cells, and then cultivated them in the cultivation dish without any control of their population or no formation of community with other cell types, and finally they were analyzed as a group regardless of their differences of cell cycles regardless of their possible differences. In contrast, on-chip cellomics technology adopted the new three step strategies: first, the cells were adopted from the community using non-destructive cell sorting procedure, then the cells were cultivated in a mocrochambers, in which cell network formation and medium environment was controlled, and finally the genome/proteome measurement in each cell was measured.

We started a series of studies on the “determination of genetic and epigenetic control processes in cells” using on-chip microfabrication techniques and cell-based analysis. To understand the meaning of genetic information and epigenetic correlation in cells, we developed an on-chip single-cell-based microcultivation method. As we can see in Figure 2, the strategy behind our method was constructive, involving three steps.

First, we purified cells from tissue one by one in a nondestructive manner [2]. We then cultivated and observed them under fully controlled conditions (e.g., cell population, network patterns, or nutrient conditions) using an on-chip single-cell cultivation chip [3–12] or an on-chip agarose microchamber system [13–20]. Finally, we did single-cell-based expression analysis through photothermal denaturation and single-molecule level analysis [21]. In this way, we can control the spatial distribution and interactions of cells.

In this paper, we explain the aims of our single-cell-based study and the single-cell-based cultivation/analysis system especially focusing on the temporal aspects of epigenetic information using single bacterium cultivation and comparison of her direct decendants.

## 2. Why On-Chip Cellomics Technology?: Cultivation Systems from “Algebraic” and “Geometric” Viewpoint

The advantage of our on-chip cellomics approach is that it removes the complexity in underlying physicochemical reactions that are not always completely understood and for which most of the necessary variables cannot be measured. Moreover, this approach shifts the view of cell regulatory processes from a basic chemical ground to a paradigm of the cell as an information-processing unit working as an intelligent machine capable of adaptating to changing environmental and internal conditions. This is an alternative representation of the cell and can bring new insights into cellular processes. Thus, models derived from such a viewpoint can directly help in more traditional biochemical and molecular biological analyses that assist in our understanding of control in cells.

**Figure 3.**
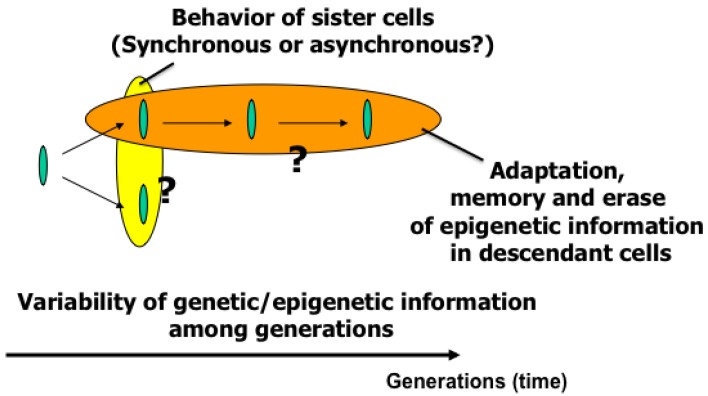
Aim of the single-cell based analysis (1): temporal aspect. In this aspect, two viewpoints of analysis should be done; one is comparing two daughter cells born from the isolated single cell, and the other is comparing the direct descendant cells for generations to evaluate whether the information/characteristics of isolated single cell can be inherited through cell divisions.

**Figure 4.**
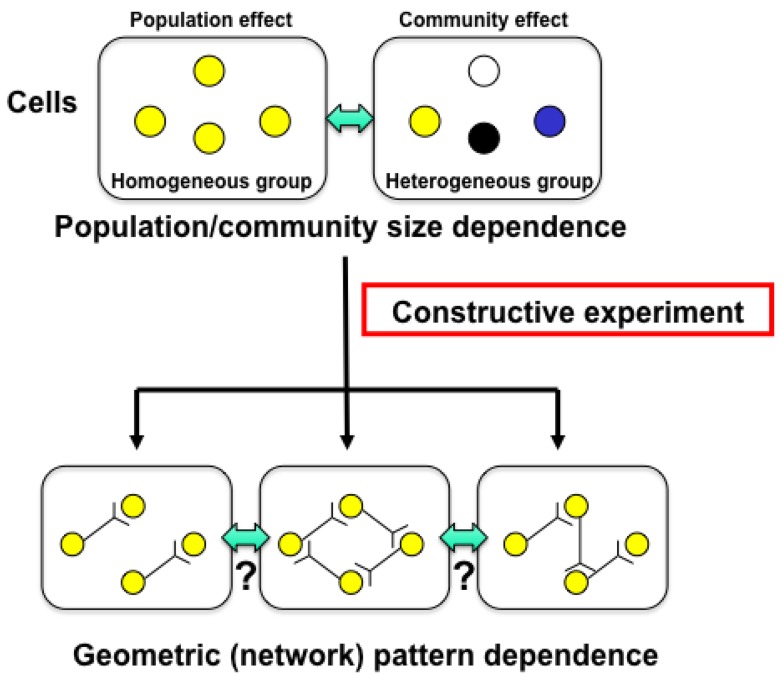
Aim of single-cell-based analysis (2): spatial (geometric) aspect. In this aspect, two viewpoints of analysis also should be done; one is population/community size dependence and the other is spatial (network) pattern dependence of group of cells.

The main purpose of the study was to develop on-chip single-cell-based cultivation and analysis systems to monitor dynamic processes in the cell. We have used these systems to extend ideas from the genetic to the genetic-epigenetic network in investigating topics like variations in cells with the same genetic information, inheritance of non-genetic information between adjacent generations of cells, cellular adaptation processes caused by environmental change, the population effect and the community effect of cells, and network pattern formation in cell groups (Figures 3 and 4). After sufficient experimental observations, we can understand the role of epigenetic information in modeling more complex signaling cascades. This field has almost been entirely monopolized by physico-chemical models, which provide a good standard for comparison, evaluation, and development with our approach. The ultimate aim of our study is to provide a comprehensive understanding of living systems as products of both genetic and epigenetic information. It would permit us to describe the phenomena occurring in cell systems sufficiently well to be able to interpret and control them.

## 3. Cultivation System For “Algebraic” Viewpoint: On-Chip Single-Cell Cultivation System For Isolated *Escherichia Coli* Cells

Phenotypic and behavioral variations from cell to cell have been observed to exist even in a genetically identical population [1,22–25]. The resulting heterogeneity in a clonal population may well be important not only for survival [24], but also for cooperation in a population that must obviously exist and work in multicellular organisms [26–28]. The mechanisms of producing phenotypic variations are explored both theoretically [29–34] and experimentally [35–37] as an intracellular noise-driven process [38]. McAdams and Arkin proposed that stochasticity in the process of gene expression could lead the substantially large difference of protein products amount, which eventually affects the switching mechanisms in individual cells in a group that select between alternative phenotypes [33]. The existence of the noise in gene expression processes was shown experimentally by van Oudenaarden and colleagues [37]. They showed that the resulting expression noise had a strong positive correlation with translational efficiency. As another example of the experiment, Elowitz and colleagues examined the contributions to overall variation from gene expression process and from other cellular components separately, showing that the noise in gene expression process did not uniquely determine the total variability [36].

These studies are based on the temporal observation of a cell group. The group based observation, however, cannot show how an individual cell produces different phenotypes and behaviors in the course of proliferation and whether phenotypes and behaviors specific to an individual cell can be inherited. Conventional techniques like flow cytometry and direct observation with a microscope provide no control over the cell-cell interactions or selection of cell type. Flow cytometry enables us to obtain the distributions of parameters like concentration, size, shape, DNA content *etc.* at the single-cell level in a group although not related to epigenetic profiles yet [39]. The problem of this method is that it cannot continuously track a specific cell's dynamics because the sample drawn from the culture is discarded after the measurement. Neither can it keep cells under isolated conditions, nor can it identify a particular cell, especially after cell division has occurred. Thus, cytometry can give us information about the average properties of cells as a summation of individual characteristics of different cells in a group, that is, how the group changes including the distribution information of individuals, but it can't give us the tracking information about how a same single cell changes. Direct measurement with a microscope of cells in solid media like cultivation plates [28,40–43] can identify individual cells, and thus, can track specific cells continuously. However, it is impossible to keep cells isolated especially after cell divisions have occurred and it is impossible to control the interactions between particular cells because the positions of the cells are fixed at the beginning of the cultivation. Thus these conventional methods are not satisfactory means of gaining an understanding of single-cell level interactions of particular cells.

As new techniques are needed to clarify the interactions between genetically identical cells, we have developed an on-chip single-cell-based microculture method exploiting recent microfabrication techniques and conventional *in vivo* techniques. The advantage of the on-chip technique is that a diverse population development out of one cell can be followed directly. To manipulate cells in microchambers, we use non-contact forces, such as optical tweezers and acoustic radiation force, which have been used to handle cells, organelles, and biomolecules on microscope specimens [2,44–49]. In this section, we briefly describe our on-chip single-cell based microculture method and explain the on-chip single cell cultivation chip.

### 3.1. System Design

**Figure 5.**
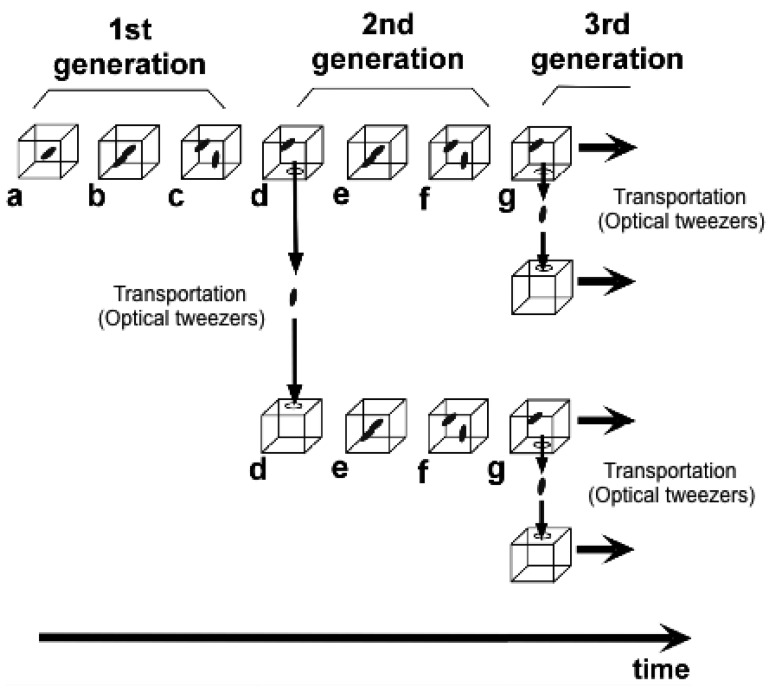
Single-cell cultivation in microchambers to measure variability in genetic information. An on-chip single-cell cultivation system enabled excess cells to be transferred from the analysis chamber to the waste chamber through a narrow channel that allowed a particular cell to be selected from cells in the microfabricated cultivation chamber with non-contact force, optical tweezers.

To understand variations in cells with the same genetic information and observe adaptation processes in cells, we need to directly compare sister or direct-descendant cells (see the viewpoints in Figure 3 and methods in Figure 5).

**Figure 6.**
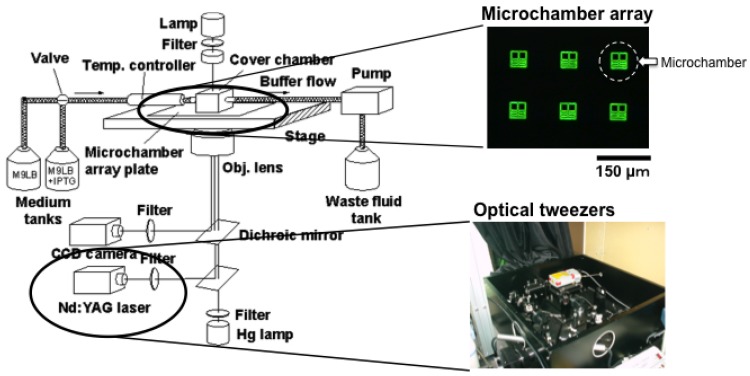
On-chip single-cell cultivation system for *E. coli* cells. The system consists of three parts; microchamber array chip with medium supply, optical microscopy with recording equipment, and optical tweezers.

Figure 6 is a schematic drawing of the entire system we used for on-chip single-cell-based analysis. It consists of a microchamber array plate (chip), a cover chamber attached to the medium circulation unit, a ×100 phase-contrast/fluorescence microscope, and optical tweezers.

**Figure 7.**
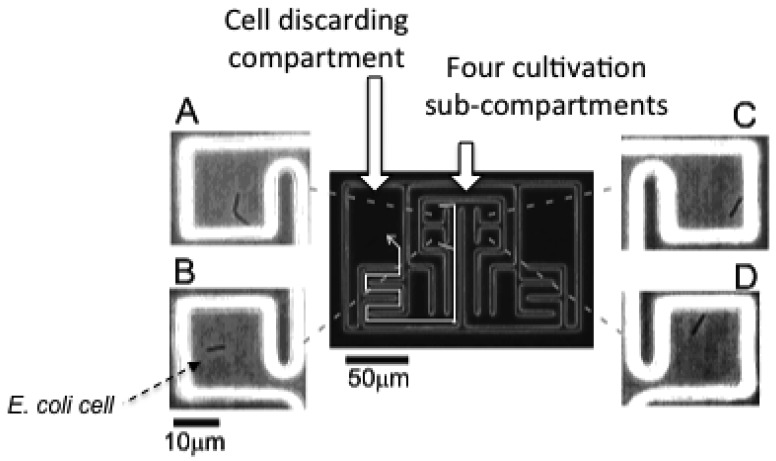
Optical micrograph of microchamber (compartment) with four sub-compartments. Each single-cell cultivation compartment has four observation sub-compartments (A, B, C, and D) at the center of the microstructure. At the both sides of four cultivation compartment, two cell discarding compartments are arranged and are connected to the observation sub-compartments with long winding narrow pathways. Enlarged views of the four cells in the four sub-compartments of the observation area can be seen.

**Figure 8.**
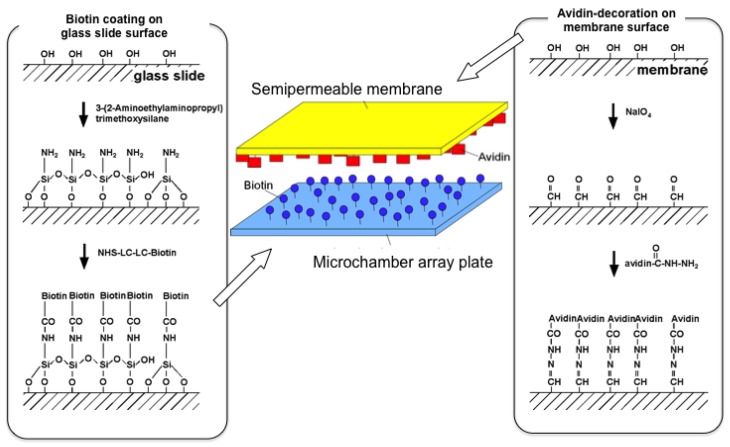
Sealing protocol for semipermeable membrane lid on chip. Semipermeable membrane is decorated with avidin, and the glass slide with biotin. After the introduction of single cells into the microchambers the semipermeable membrane lid sealed the top of the microchambers with avidin-viotin bonding to prevent escape the isolated cells from mcrochambers.

The microchamber array is the microfabricated structure on the glass slide made of thick photo-resist SU-8-5 (Microlithography Chemical Corp., Newton, MA, USA) (Figure 7). The height of the microchamber array is 5 μm, in which the cells are enclosed. The microchamber array is sealed with a semipermeable membrane to prevent the cells escaping from it. The semipermeable membrane is decorated with avidin and the glass slide with biotin to ensure the seal is tight (Figure 8). With these decorations on the membrane and slide, it is possible to observe cells in the microchamber without them escaping. The microchamber (compartment) is composed of two main parts. The first is the observation area, which has four sub-compartments in it at the center of the microchamber. Each sub-compartment has a volume of 20 × 20 × 5 μm. The second part includes the discarding areas at both sides of the four-room sub-compartments.

The first four direct-descendant cells derived from a isolated single cell were placed in one of the four sub-compartments individually to keep them isolated. The excess descendant cells were transferred to the two large discarding areas along the white arrow with the optical tweezers through the narrow winding path as shown in Figure 7.

As we can see in the micrographs, only one cell is enclosed in each of the four sub-compartments of the observation area under isolated conditions. Four specific cells in the four sub-compartments were simultaneously observed without any disruption by the other cells and without leaving the field of view of the microscope.

Optical tweezers were introduced to enable non-contact handling of the cell specimens. An Nd:YAG laser (wavelength = 1,064 nm, T20-8S, Spectra Physics, Santa Clara, CA, USA) was guided to the ×100 phase contrast objective lens (UplanApo, Olympus, Tokyo, Japan) in a phase contrast microscope (IX-70, Olympus, Tokyo, Japan) as the light source for the optical tweezers, which are widely used in handling micron-sized particles and biomaterials [44–49]. We used it in the system in our protocol to transport particular cells within the microchamber.

The medium circulation unit utilized a glass box with a volume of 1 mL that had two branches. It was mounted on the microchamber array chip and a fresh medium buffer was always circulated in the glass box through the two branches at a rate of 1 mL/min with a peristaltic pump. The bottom of the glass box was open and the condition of the medium around the cells could be constantly maintained by buffer exchanges through the semipermeable membrane.

The whole microcultivation part was placed in a thermo control cage (IX-IBM, Olympus, Tokyo, Japan) to maintain the temperature at 37 °C throughout observation. The observation images were taken with a CCD camera (CS230, Olympus, Tokyo, Japan) and recorded on digital video cassette. These were analyzed on a personal computer (PCV-R73K, Sony, Tokyo, Japan).

### 3.2. Differential Analysis of Sister Cells with Identical Genetic Information and Experience

**Figure 9.**
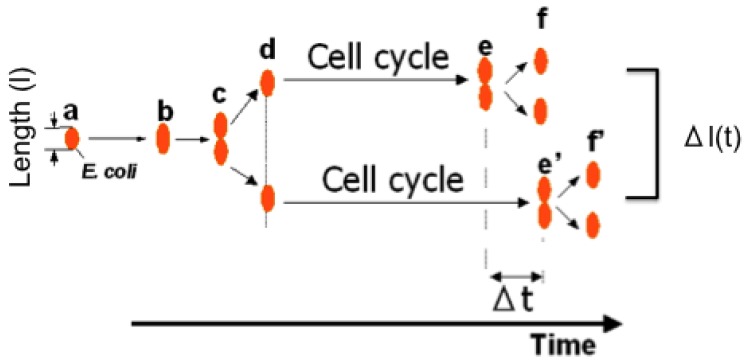
Differential analysis of sister cells. During on-chip cultivation, both the difference of interdivision time (cell cycle) and lengths at time (t) of sister cells were compared.

To investigate non-genetic variability in the division cycle and growth of single cells, we first compared the growth and division times for pairs of *E. coli* daughter cells under isolated conditions using the on-chip single-cell cultivation system we just described in [3,5] (see Figure 9).

**Figure 10.**
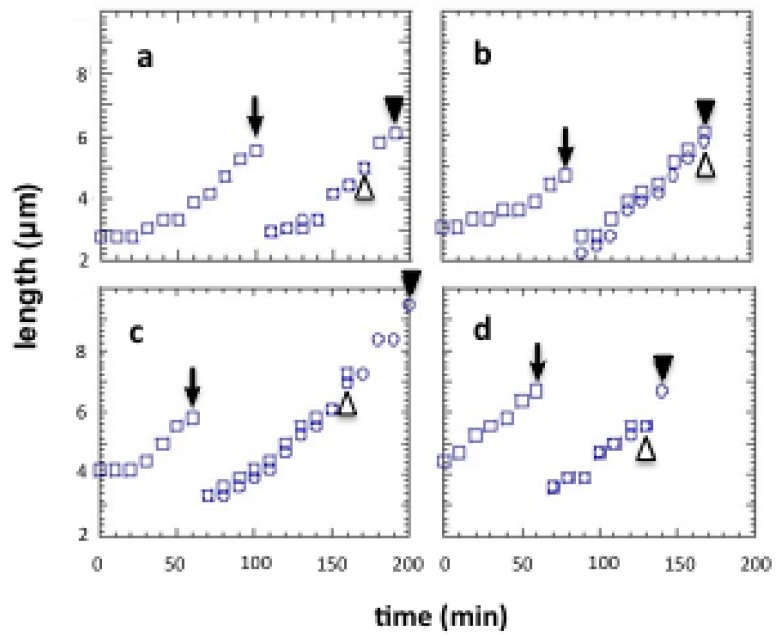
Time course growth for isolated individual *E. coli* and two daughters. After on-chip cultivation started, an isolated single cell (mother cell) grew in the microchamber, and finally divided into two daughter cells (see the arrows). Although the newborn daughter cells grew synchronously in the same manner, they divided into granddaughter cells at different times, (see filled arrowhead and open arrowhead in the graph representing the division times of each of sister cells, respectively).

In this experiment, we used *E. coli* strain JM109 in a minimal medium, M9 (4.5 g/L KH_2_PO_4_, 10.5 g/L K_2_HPO_4_, 50 mg/L MgSO_4_·7H_2_O, pH 7.1) containing 1 × 10^−5^ % (w/v) of glucose at 37 °C. Figure 10 shows the four typical examples of the differential analysis of sister cells within the four compartments in a microcultivation chip. Hence the growths and cell divisions in Figure 10(a–d) was observed simultaneously in the same circumstances. For example, after on-chip cultivation started, an isolated single cell (mother cell) grew in the microchamber after the resting of growth from 2.8 μm to 5.6 μm in 90 min, and finally divided into two 2.8-μm daughter cells (see Figure 10(a)). Although the newborn daughter cells grew synchronously in the same manner, they divided into granddaughter cells at different times, *i.e.*, 70 min and 90 min (see arrowheads in graph). The three other examples (Figure 10(b–d)) show that even though the growth of the mother cell and her daughter cells seems to have no significant correlation, the growth of two daughter (sister) cells from the same mother cell seems to be quite similar. In contrast, the division times for daughter cells of the same length (Figure 10(a,c,d)) were not synchronous. In Figure 10(b), on the other hand, the division time and cell growth tendency of two daughter cells were synchronous even though they were born after unequal divisions of the mother cell. These results indicate that variations in cell growth and cell division may not be closely correlated and that cell division time is independent of genetic identity and cell size.

**Figure 11.**
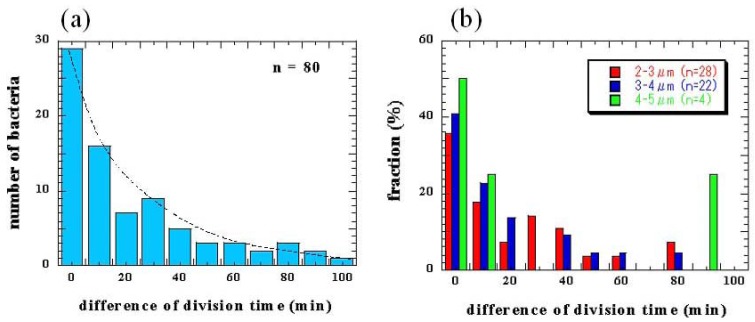
(**a**) Differences in division time for two daughter cells of same mother cells (n = 80 pairs), and (**b**) initial dependence of division time differences on length.

The division time differences between two daughter cells from the same mother cells were also measured (Figure 11). Although sister cells are thought to have the same DNA and chemical components as their mother's cells, the results revealed only 36% of daughter cells divided into granddaughter cells within a 10-min difference of period even when they started at the same cell lengths (Figure 11(a)). The dependence of division time differences for newborn daughter cells on length was also evaluated and the time distribution was similar regardless of the initial length (Figure 11(b)). These results also indicates that variations in cell division may not depend on DNA mutation or the initial cell size.

The initial dependence of variations in cell growth and division on length was also evaluated. The ratio of the final length of these cells and their initial length seems to be independent of the initial length, about 170%, when it is longer than 3 μm. The speed of growth of cells also has no significant dependence on the initial length.

It has been already well described for *E. coli* and also for a few other bacterial strains that the cell division time itself is nearly constant (deterministic) but the time point in cell cycling for the division decision can be very different. This is the pre-D time (a stochastic event) and its duration is determined by micro-environmental conditions, and probably also by epigenetics including actual cell sizes [50].

### 3.3. Differential Analysis of Direct-Descendant Cells with Identical Genetic Information and Experience

**Figure 12.**
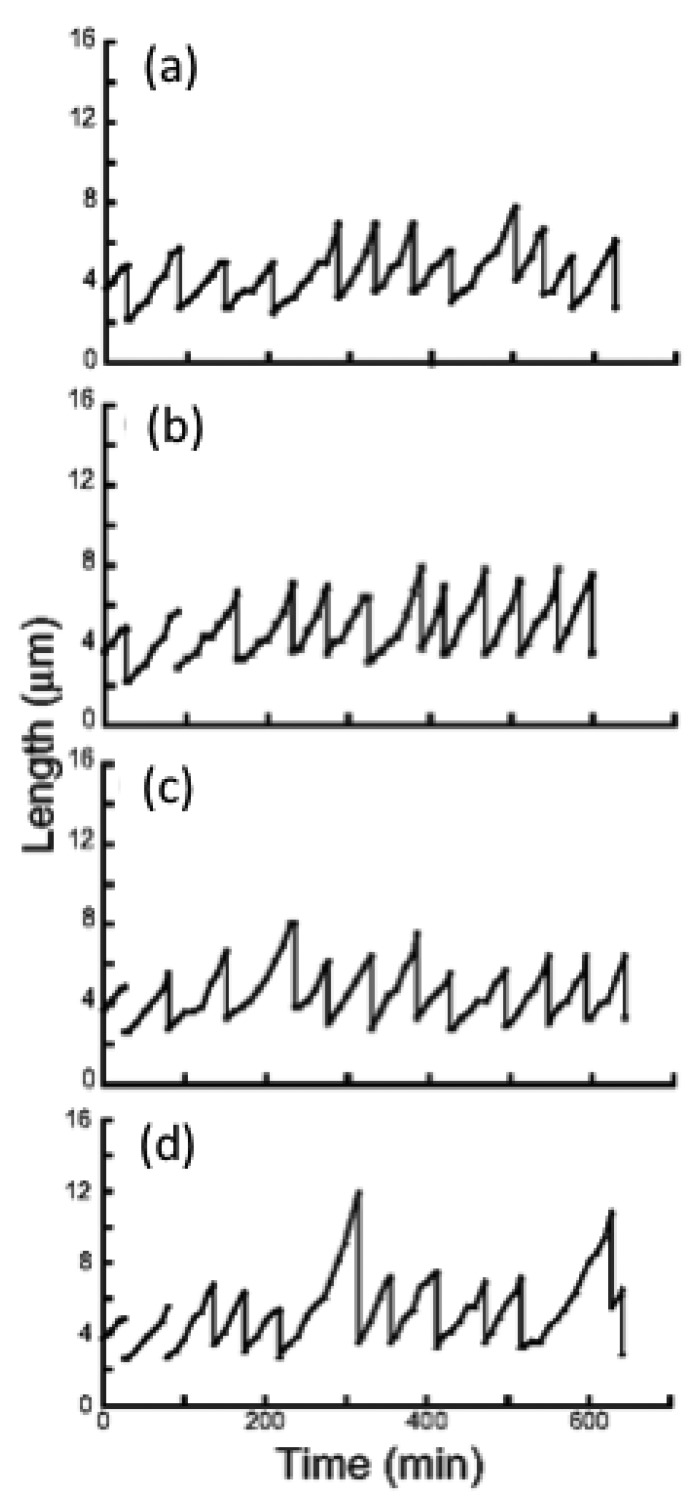
Temporal variations in cell lengths of individual cells and their direct descendants. (**a**) to (**d**) indicate growth and division patterns for four cells born from a single cell and isolated into the four sub-chambers A to D in Figure 7.

We next examined whether the characteristics of direct descendants of an isolated single cell could be inherited under isolated conditions using the on-chip single-cell cultivation/analysis system [4,6]. Figure 12 plots temporal variations in cell lengths of individuals and their descendants. Figure 12(a–d) indicate growth and division patterns for four cells born from a single cell and isolated into the four sub-chambers A to D in Figure 7.

**Figure 13.**
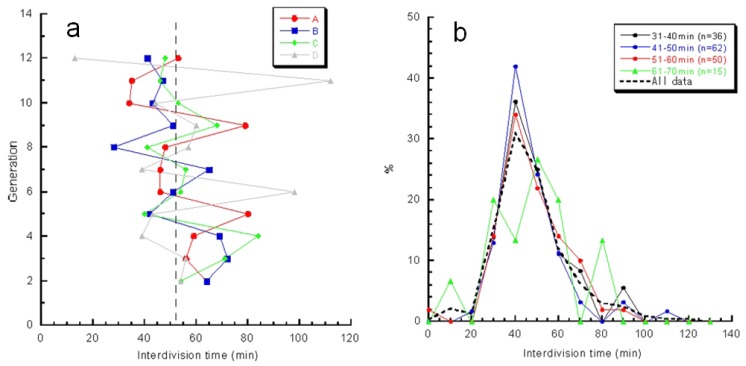
Genetic variations in direct descendant cells of *E. coli.* (**a**) Interdivision time of generations of direct descendant cells of four sister cells. (**b**) Interdivision time dependence of populations of direct descendant cells in (a).

Figure 13(a) also plots variations in interdivision times for consecutive generations of other isolated *E. coli* cells derived from a common ancestor. The four series of interdivision times varied around the overall mean value, 52 min (dashed line); the mean values of the four cell lines a, b, c, and d were 54, 51, 56 and 56 min, indicating rather small differences compared with the large variations in the interdivision times of consecutive generations. These results support the idea that interdivision time variations from generation to generation are dominated by fluctuations around the mean value, and this was evidence of a stabilized phenotype that was subsequently inherited. To explore this idea further, we examined the dependence of interdivision time on the interdivision time of the previous generation. We grouped all interdivision time data into four categories and calculated their distributions (Figure 13(b)). A comparison of these distributions revealed that they were astonishingly similar, suggesting that there was no dependence on the previous generation. That is, there was no inheritance in interdivision time from one generation to the next.

### 3.4. Adaptation Process for Sensor Proteins in Cells Caused by Environmental Changes

We then modified this on-chip single-cell cultivation/analysis system to simultaneously measure the sensor-protein dynamics and motility of identical single cells for several generations.[8] This technique revealed the potential of combining the microfabrication technique (single-cell cultivation technique) and molecular biology (single-molecule observation).

*E. coli* cells are able to respond to changes in environmental chemo-effector concentrations through reversing their flagellar motors [51,52]. Attractants (such as aspartate and serine) promote counterclockwise rotation of the flagella, resulting in a smooth swimming action, whereas repellents (such as phenol and Ni) promote clockwise rotation, resulting in tumbling. These responses are mediated by membrane-bound, methyl-accepting chemoreceptor proteins (MCPs). Immunoelectron microscopy revealed that MCP-CheW-CheA complexes are clustered *in vivo*, predominantly at the cell poles [53], and merely weaker lateral clusters could be observed [54,55]. Polar-localization changes have been expected according to environmental conditions, whereas no evidence concerning the dynamics of localization-changes has been reported.

**Figure 14.**
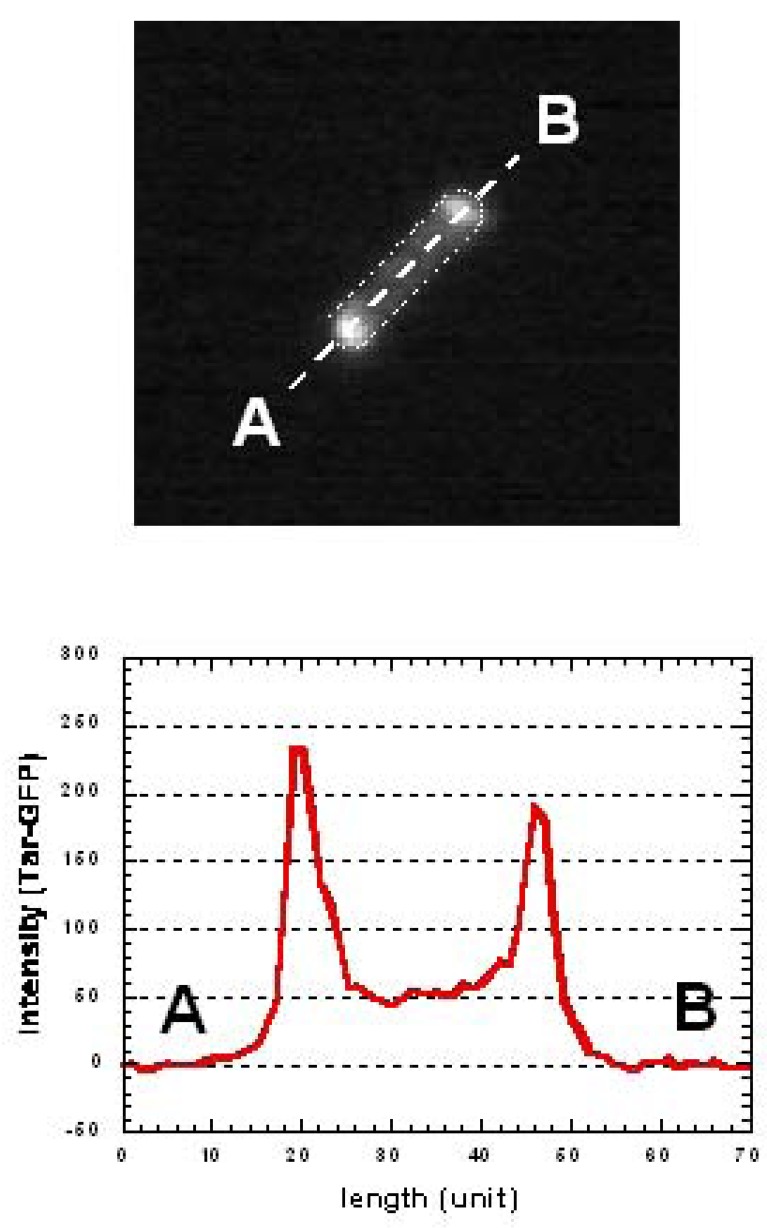
Tar-GFP localization in *E. coli* cell. Upper fluorescence image shows the localization of GFP-tar proteins at the both poles in a *E. coli*. Lower graph shows the histogram of GFP localization profile from point A to B (see upper image) indicating fluorescence localized at poles.

Conventional group-based experiments do not allow the process of MCP clustering and the effect its change has on consecutive generations in individual cells, which is essential in estimating the changes occurring during the alternation of generations. To understand epigenetic processes such as adaptation and selection, both the protein-dynamics and the cell-dynamics of particular single cells should be observed continuously and simultaneously for several generations. We used assayed intracellular proteins tagged with green fluorescent protein (GFP) to measure the localization-dynamics of expressed proteins (Figure 14).

**Figure 15.**
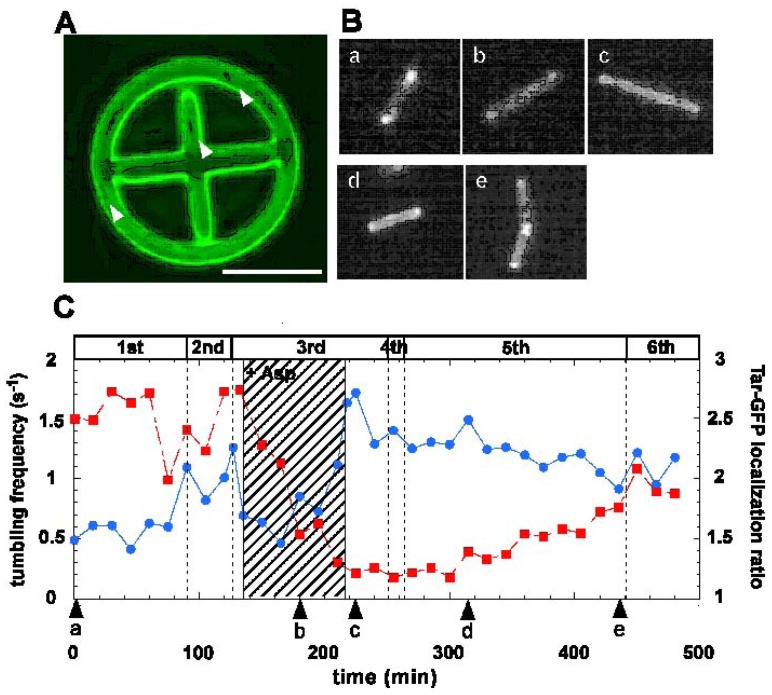
Simultaneous observation of Tar-GFP localization and motility in identical *E. coli* cell for several generations. When the cultivation started, the tar-localization ratio (red squares) was 2.5 and the tumbling-frequency (blue circles) was 0.5 (s-1) [(a) in Figure (A), and arrowhead “a” in graph in Figure (C)]. After the second cell division had occurred, a minimal medium containing 1 mM of aspartate was applied to the third generation cell (135 min after microcultivation). After the attractant was added, tumbling-frequency (filled circles in graph) decreased immediately compared to the previous generation. Localization of the aspartate sensitive sensor protein at two poles in Escherichia coli (filled squares) also decreased quickly by half to 45 min following the change of medium (Figures B(b) and C(b). Finally, after 80 min of stimulation with the aspartate, the localized tar had diffused completely. Then, the aspartate was removed from the cultivation medium and the cells were cultivated further to enable the recovery of tar-localization dynamics to be measured (Figures B(c) and C(c)).

We modified the shape of the microchambers into a wheel to measure the time course for motility (Figure 15A). In the experiment, we first placed a single bacterium in the microchamber and isolated it in the wheel region so that it could swim along the track seal with the semi-permiable membrane lid on the microchamber. Then, the bacterium running around the circle structure was continuously monitored by measuring the tumbling frequency and protein-localization dynamics. When the cell divided into two daughter cells, one of these was picked up with the optical tweezers, transported to the axle area, and continuously confined in this region to stop its growing. The bacterium was chemically stimulated by adding aspartate (nutrition for *E. coli*) into the medium.

After the first change of medium, it took more than three generations to recover the original pattern of tar localization (Figure 15B(d,e) and 15C(d,e)). However, the frequency of tumbling remained higher than the former generations. This may indicate that tar-localization requires more time to form than to diffuses. Such asymmetric reversibility in protein localization may contribute to cell phenomena being inherited caused by environmental changes. It also suggests the possibility that change in tar localization can be inherited by descendant cells and this can affect their motility and therefore their phenotype.

### 3.5. Origin of Individuality of Two Daughter Cells during the Division Process Examined by the Simultaneous Measurement of Growth and Swimming Property [56]

Detecting changes in the swimming behavior of a particular line of growing cells seems feasible but has been challenging many scientists because it is experimentally difficult. It is impossible to follow direct descendant cells in a test tube, and even if they were identified they could not be observed continuously as they grow. Clearly identifying transient time-course changes in particular function is difficult, and they must be investigated separately because they might provide information about the relation between growth and motility but not about when the individuality of a particular cell arises.

We have overcome these experimental limitations by developing a system that measure growth and motility simultaneously. Its single-cell-based dual-recording feature has made it possible to directly compare the cellular growth and motility of a cell with that of its direct descendants.

**Figure 16.**
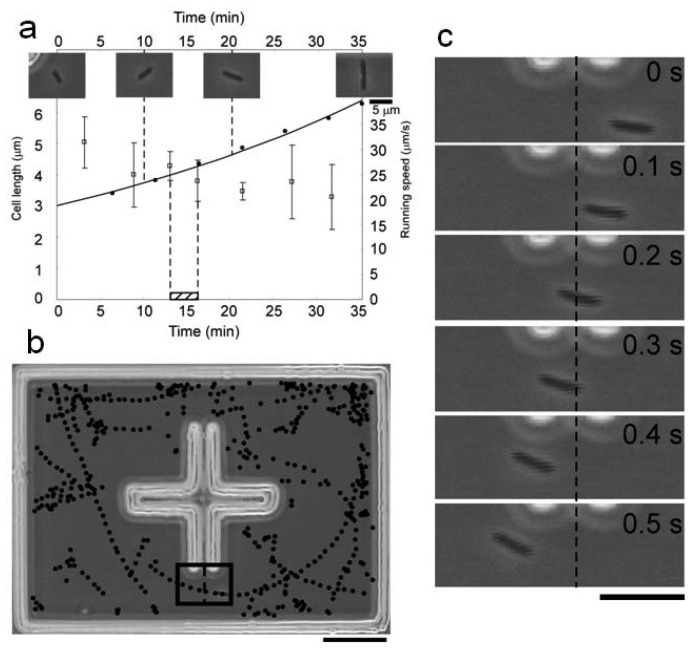
Typical example of simultaneous measurement of growth and motility. (**a**) Time course of single *E. coli* growth and motility for one cell cycle (t = 0 to 35.3). Cell length (filled circles with a fitted exponential growth curve) steadily increased as the running speed (open squares with S.D.) decreased throughout the cell cycle. Photographs show the morphology of the cell at each corresponding time point. (**b**) Swimming path of the cell. Black dots are recognized positions of the cell by the computer analysis program for the hatched time range shown in (a) (t = 13.0 to 16.2). Bar, 20 μm. (**c**) Photographs of the signified region in (b) at successive 0.1-s frames. The cell was swimming through the region from right to left. Bar, 10 μm.

A typical result of simultaneous measurement of cell length and running speed is summarized in Figure 16. Figure 16(a) is a record of cell length and running speed for one generation, from the birth of a cell to that of its daughter cells. Throughout its cell cycle the cell steadily elongated exponentially. Its running speed, on the other hand, gradually decreased throughout the cycle. The running speed was calculated on the basis of the recorded positions of the cell. By plotting these positions we can also generate the swimming path of the cell for an arbitrarily defined time range (Figure 16(b)). Each position was determined by an image analysis program driven in real time with 0.1-s resolution. The program recognized the positions of the cell based on a successive series of high-resolution images (Figure 16(c)).

**Figure 17.**
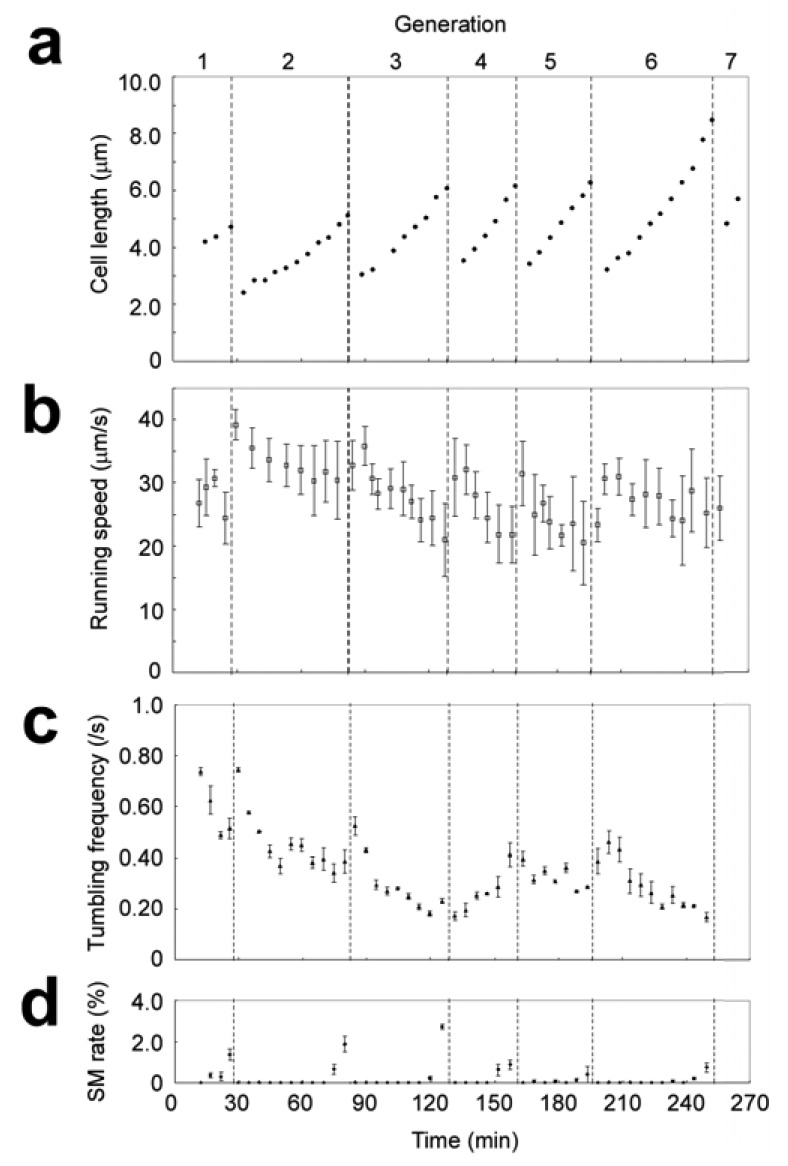
Simultaneous measurement of growth and motility for successive generations. Time course of single *E. coli* growth and motility were monitored by the measurement of (**a**) cell length, (**b**) running speed (mean ± S.D.), and (**c**) tumbling frequency (mean ± S.E.). Vertical dashed lines correspond to the timing of cell divisions. (**d**) Prolonged pausing found in the final stage of cell cycles. Duration of pausing per each tumbling was averaged in a time window of 1 min and plotted as a function of time for the generations 2 through 6 with the timing of cell division (vertical solid lines) as well as the initiation of cell constriction (vertical dashed lines). Compared to the mean values before constriction (horizontal solid lines, mean; horizontal dashed lines, S.D.), the duration tended to increase in the final stage of cell cycles.

By combining the repeated isolation procedure with this analysis, we can compare the growth and motility of an isolated single cell with the growth and motility of its direct descendants. Figure 16 is an example of simultaneous measurements for seven generations. The cell length smoothly increased within each cell cycle (Figure 17(a)). The running speed decreased throughout each cell cycle (Figure 17(b)). Within each cell cycle the plots of running speed versus generation time tended to form smooth curves, but the slopes of those curves differed between one generation and another. Tumbling frequency fluctuated between generations and did not show any constant increase or decrease (Figure 17(c)).

Careful observation revealed that a pre-divisional cell after constriction showed characteristic movement (movies available as Supplemental Material). It can be characterized by a longer duration of pausing in motion: typically 10^−1^–10^0^ s, which is about ten times longer than that caused by ordinary tumbling (10^−2^–10^−1^ s). We named it *prolonged pausing* in contrast to ordinary tumbling. To analyze it in a quantitative manner, we measured the duration of each pausing in motion and averaged in a 1-min time window (Figure 17(d)). The duration of pausing remained relatively constant before cell constriction, but it tended to increase after constriction to the level beyond the deviation from the pre-constriction value (generation 5 seemed to be an exception). The increase in pausing duration appeared only after cell constriction, reflecting that only pre-divisional cells exhibit prolonged pausing.

The results of this on-chip measurement were consistent with the results of conventional methods. The measured running speed and tumbling frequency were both acceptable when compared to those that have already been reported for the wild-type strain (25 μm/s and 0.53 s^−1^) [57]. This means that the microchambers confining the cells did not affect the swimming behavior, and that the on-chip measurement was compatible with other conventional methods.

More important is that we have measured growth and motility separately but simultaneously. Conventional methods cannot deal with both of them because growth needs to be watched for a longer term, whereas motility requires close observation for a shorter term. Moreover, conventionally obtained results describe only the average characteristics of a group of cells. The improved on-chip single-cell cultivation system that copes with swimming bacteria has overcome these limitations. We have shown a direct relation between growth and motility: cell-cycle dependence of swimming behavior.

We found prolonged pausing that appeared exclusively in the final stage of cell cycles, after the initiation of cell constriction. This prolonged pausing in pre-divisional cells poses an important question about the emergence of individuality of the subsequent daughter cells.

Mother cells and daughter cells are usually defined by cell division: a physical process that divides the cell body into two newborn ones. To complete cell division and to become two cells, however, a cell must spend some time preparing at molecular and cellular levels. Thus there must be a transient state in which two distinct control systems coexist in a pre-divisional cell. These two systems would independently affect the intracellular mechanism for the whole-cell moving behavior.

Prolonged pausing, which appeared between the initiation of cell constriction and the physical separation into two new cell bodies, is probably reflecting this behavior. After the initiation of cell constriction, cellular contents are segregated into two daughters-to-be by an internal structure called a septum. During the segregation process it is likely that switching in one daughter-to-be cannot be synchronized smoothly with that in the other daughter-to-be. This asynchronous state may have been observed as prolonged pausing. Because of its characteristic movement it differs from pausing of antibiotic-treated filamentous cells, shown to be equivalent to ordinary tumbling of normal-sized cells [58]. Rather we hypothesize that the observed prolonged pausing reflects the coexistence of two distinct control systems within a mother cell; that is, individuality emerges after a single cell initiates constriction and before it gets physically separated into two new cell bodies.

Individuality has also been discussed from the viewpoint of bacterial swimming behavior by Spudich and Koshland [1]. They reported that cellular individuality in bacteria is rather steady throughout their cell cycles. Although the phenomenon in this report looks like the opposite from our results, it does not contradict this study. Because they regarded cell length as an index of cell cycles, and they neglected the fluctuation of synchrony between cell length and cell cycle. Since even direct descendant cells vary in length [6], we cannot infer that different cells of the same cell length are at the same stage of their cell cycles. The prolonged pausing we have found here was probably hidden by this large variation, and was revealed by the long-term single-cell monitoring.

### 3.6. Asynchrony in the Growth and Motility Responses to Environmental Changes by Individual Bacterial Cells [59]

Various physiological states of bacteria are related to their survival strategy under environmental stresses. One example of the phenotypic diversity is a non-growing but metabolically active state called quiescence, in which cells channel available metabolic resources toward particular cellular functions, such as the expression of specific genes [60].

In this subsection, we have studied how the growth and motility of a single cell change in response to a change of the external nutrient condition. Simultaneous monitoring of growth and motility with an on-chip single-cell cultivation system revealed asynchrony in the growth and motility responses to nutrient starvation. Cell growth stopped quickly after starvation began, whereas cell motility was first maintained for several hours and then gradually lost. Discussing these results, we consider energetics within an individual cell, the flagellation state of starved cells, and possible mechanisms responsible for the observed response asynchrony.

An *Escherichia coli* strain AW539 was transformed with a plasmid coding a GFP-tagged aspartate receptor for this study. This study demonstrated the feasibility of long-term, transgenerational observation of single-cell growth and motility with the sequential use of multiple medium conditions of choice. We applied nutrient condition changes to an isolated single cell to measure the response of its movement and growth after the environmental changes. In each experiment a motile cell was chosen and then continuously monitored, swimming freely but only within its chamber. Transgenerational observation was achieved by using optical tweezers to repeatedly remove sister cells produced by cell division. During the repeated removal the laser power was increased so that the unwanted cells were “killed” and permanently fixed to the dead-ends of the chamber. The isolated condition was maintained during medium exchange. The series of abovementioned procedures provided results presenting the time-course change of cell length and running speed for multiple generations.

**Figure 18.**
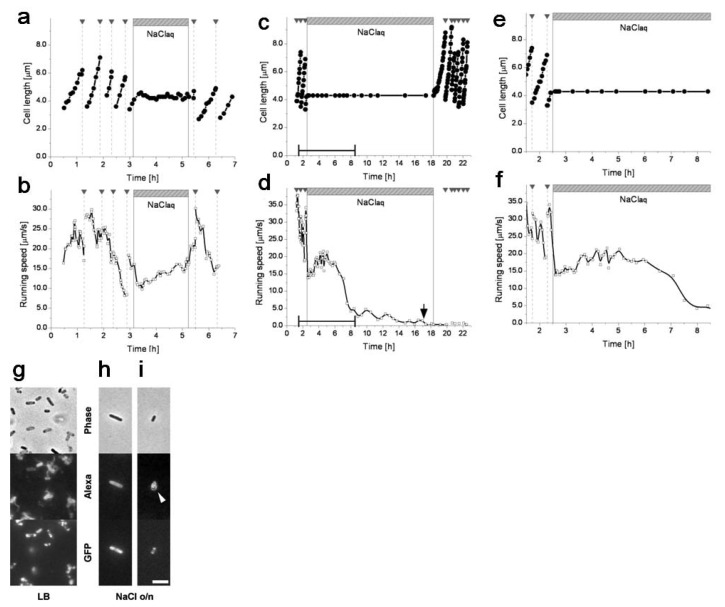
The effect of 2-h starvation on the single-cell growth and motility (**a,b**). The medium was changed from LB to NaCl solution (hashed) and then back to LB again. (a) The growth curve of a single cell and its direct descendants. (b) The speed curve of the same cells. The triangle symbols with vertical dotted lines indicate the timing of cell division. The effect of 16-h starvation on the single-cell growth and motility (**c**–**f**). The medium was changed from LB to NaCl solution (hashed) and then back to LB again. (c) The growth curve of a single cell and its direct descendants. (d) The speed curve of the same cells. The triangle symbols indicate the timing of cell division. The arrow in (d) indicates when the cell completely lost its motility and started to show Brownian motion. (e) A magnified graph of (c) showing the region between 1.5 and 8.5 h. (f) A magnified graph of (d) showing the region between 1.5 and 8.5 h. Flagellation state of starved cells (**g**–**i**). (g) Control cells cultured in LB medium. Most of the cells had multiple flagella. (h,i) Cells that experienced overnight (20 h) nutrient starvation. Almost all were deflagellated like that shown in (h), but a very few had a short single flagellum (indicated by the arrowhead) like that shown in (i). Phase: phase-contrast images. Alexa: fluorescent images of Alexa 546 that labeled flagella and the entire cell body. GFP: fluorescent images of membrane chemoreceptor protein Tar-GFP for the identification purpose. Note that the fluorescent images are false-colored. Bar, 5 μm.

The simultaneous observation of cell growth and motility revealed asynchrony between the two functions when a single cell respondsed to nutrient starvation (Figure 18). The cell cycle of the single cell was first checked in the constant LB (nutrient-rich) medium condition. During each of the first few generations, the growth curve increased monotonically and the speed curve showed a general tendency to decrease. After a few generations, the LB medium was completely exchanged for 0.9% NaCl aqueous solution (nutrient-free) so that the cell started experiencing nutrient starvation. Cell growth stopped abruptly (in 10–20 min), after which cell length (and its shape and volume) remained the same. On the other hand, the cell remained motile with occasional tumbling during starvation. The running speed initially dropped in the first 5 min but then started to increase slightly. After the two-hour starvation period, we exchanged the saline medium for the nutrient-rich LB medium. The cell resumed its growth in about 10 min and then proliferated normally. The running speed increased sharply (in ≈5 min) before the first cell division, and then the cell exhibited the same tendency of decrease in speed that it exhibited before starvation.

Whereas the cell maintained its motility during starvation, it lost its motility during an extended starvation period (Figure 18(c–f)). When the nutrient-free medium replaced the nutrient-rich medium, the initial response was the same as that seeing during short-term starvation: the cell stopped growing but kept moving, and the running speed dropped within 5 min but then started to increase slightly. A few hours after the medium was exchanged, the running speed reached a local maximum and started decreasing. During this slowing-down process the cell did not swim as smoothly as before, frequently stopping and rotating. Eventually the cell lost its motility and started to demonstrate Brownian motion (indicated by the arrow in Figure 18(d)). Motility was not recovered after the reintroduction of LB medium, but growth and proliferation resumed.

Because growth-suspended cells remain motile, we think that cellular activity gets channeled into selected functions. Like non-growing but metabolically active quiescent cells [60], starved cells might use resources normally allocated to growth and division to maintain the transmembrane proton gradient that drives flagellar rotation. This could be related to a foraging strategy in *E. coli* in that the starving cells would be taking a risk to increase their likelihood of survival by redistributing available energy [61,62].

The molecular basis of the maintenance of motility remains to be revealed. Related physiological factors include pH, osmotic pressure, and chemotactic attractants present in the medium. For example, gene regulation is known to be affected by external pH [63]. The effects of these factors can be investigated individually by appropriate combinations of medium conditions.

What is behind the observed response asynchrony? Or, what is behind the apparent irreversibility in cell motility? Mutations are usually unlikely to alter the genetic information of direct descendant cells, since the per-genome mutation rate in *E. coli* is only 0.0025 per genome replication [64], but the mutation rate in extremely severe environments could be tuned considerably higher as a stress response [65]. The apparent irreversibility might also be due to epigenetic mechanisms that either work within an individual cell or depend on the presence of other cells. Intercellular communication such as quorum sensing [66] or contact-dependent regulation [67] could be the mechanism.

### 3.7. Quantitative Evaluation of Cell-to-Cell Communication Effects in Cell Group Class Using On-Chip Individual-Cell-Based Cultivation System [68]

Cells possess the ability to adapt their phenotypes through cell-to-cell communication, which underlies the cooperative behavior among interacting cells in a group [26–28,69–75]; the studies on cell-to-cell communication are therefore of great importance to reveal how cooperative behavior and multicellularity emerge in the class of cell group. To understand the role of a particular style of cell-to-cell communication from the temporal and spatial aspect, the effects from all the other possible styles must be carefully excluded; thereby the sole effect from the specific style of cell-to-cell communication on cellular phenotype becomes measurable and understandable. Cell-to-cell communication can be divided at least into two essentially different styles: communication through diffusible signals and that through cell-to-cell direct contact. Effects from these two styles on cellular phenotype should be separately examined by observing the responses of cells to the imposed style of communication. The conventional methods of cell observation, however, are unable to distinguish between these two styles; cells interact with each other extensively in complicated manners in normal cell cultures [67,73,76]. Therefore, a new method to separately control the style of cell-to-cell communication is desired to take a logical approach to the understanding of the exact role of cell-to-cell communication on cellular phenotype.

For the purpose of developing a new cell observation method in which the style of cell-to-cell communication is controlled, we came up with an idea to apply a kind of microfluidic system called an “on-chip single cell cultivation system”. In this system, cells are enclosed in “microchambers” and observed continuously for many generations under the isolated and strictly controlled environmental conditions. Cell-to-cell direct contact can be avoided by use of optical tweezers. Moreover, the medium conditions surrounding isolated cells can be changed at arbitral timings; the responses to the changes of medium conditions can be investigated at the individual cell level. Therefore, by extracting a medium from a cell culture at high cell density and imposing it on isolated cells, the effect of cell-to-cell communication only through diffusible signals is measurable.

Thus we have examined the sole effect of the cell-to-cell communication through diffusible signals under the conditions where the cell-to-cell direct contact is strictly avoided, using *E. coli* (EJ2848) as a model organism. We thereby show how individual isolated bacterial cells change their cellular states in response to diffusible signals and that the populations' coordinated entries into stationary phase are explainable from the characteristics of single cells' responses to the diffusible communication signals.

All the cell cultivations in the preparation were done at 37 °C with M9 minimum medium (Qbiogene) supplemented with 0.2% (w/w) glucose and 1/2 MEM amino acids (Invitrogen). For acquireing stationally state medium, first, a 5-μL of glycerol stock of EJ2848 was inoculated in a 1-mL M9 medium, and cultured overnight at 37 °C by shake. A 100-μL of the full growth culture was diluted in 40-mL M9 medium and cultured by shake for 20 hours. Note that the cultures under the conditions presented here reach stationary phase in approximately 10 hours after the dilution. Then, the stationary phase culture was centrifuged at 2,500×g for 10 min. The supernatant was filtered by a 0.2-μm syringe filter, thereby we obtained the stationary phase medium.

As a medium containing communication signals, we extracted the solution from the stationary phase cultures of *E. coli* cultivated in M9 minimum medium (Qbiogene) supplemented with 0.2% (w/w) glucose and an amino acids mixture (1/2 MEM amino acids, Invitrogen). The stationary phase medium was prepared from the cultures that entered stationary phase approximately 10 hours before the extraction. Cells in the mid-exponential phase of batch cultures were loaded into the microchambers shown in Figure 7. The microchamber was made of a thick negative photo-resist with high aspect ratio, SU-8-5 (MicrochemCorp), whose height was adjusted to 5 μm in the fabrication process. Cells in any of the four rooms of microchambers in the observation area were continuously observed by maintaining them under the isolated conditions by use of optical tweezers. The cells removed from the observation area were transferred and enclosed in the discarding areas at the both sides of the microchamber. The microchamber was covered by a cellulose semipermeable membrane (M.W. 50000, SpectraPor^®^) to avoid the escape of the cells. The membrane was decorated with streptavidin whereas the glass slide was decorated with biotin, making the bond between the membrane rid and the glass slides strong. The media were flown constantly at the rate of 1 mL/min. The medium in the microchamber can be exchanged rapidly by diffusion with that flowing above the membrane rid because the height of microchamber (*h*) was as low as 5 μm; the exchange speed of glucose (diffusion constant: *D* = 6.7 × 10^−10^ m^2^ s^−1^), for instance, is calculated to be 19 s (*h*^2^/2*D* = 19 ms).

**Figure 19.**
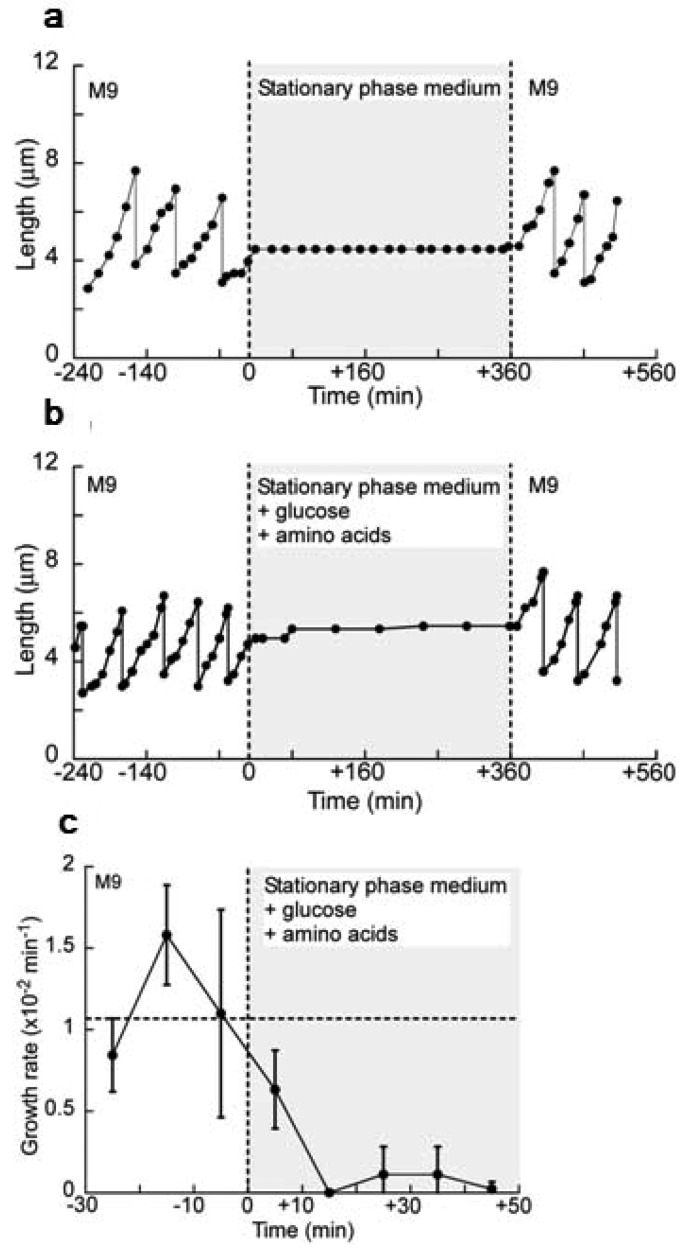
Response of exponentially growing cells to stationary phase medium. (**a**) Response to stationary phase medium without the supplements of glucose and amino acids. The medium was exchanged from M9 medium to the stationary phase medium in 240 min since the beginning of the observation. The medium was also exchanged from the stationary phase medium to M9 medium in 360 min since the previous medium exchange. (**b**) Response to stationary phase medium with the supplement of glucose and amino acids. (**c**) Growth rate transition around the medium exchange from M9 medium to the nutrient-supplemented stationary phase medium. The transition of the growth rate average of every 10 minutes of the four isolated single cells around the medium exchange was plotted. The error bars show the standard deviations of the growth rates of the four cells. The horizontal dotted line denotes 1.07 × 10^−2^ min^−1^, which is the averaged growth rate over one hour before the from-M9-to-stationary medium change.

With this system, we examined how exponentially growing isolated *E. coli* cells respond to the stationary phase environment, which should contain abundant diffusible communication signals. First, a cell was cultured in a microchamber in the constant flow of M9 medium using the on-chip single cell cultivation system. Then, the flowing media was exchanged from M9 medium to the stationary phase medium rapidly 240 min after the observation started by flushing the stationary phase medium for 30 s at the rate of 30 mL/min. The flow rate was maintained at 1 mL/min thereafter. Figure 19(a) shows one example of the response of a single cell to the stationary phase medium. The cell stopped its growth and division instantly in response to the stationary phase medium. Moreover, it showed no growth and division in the stationary phase medium. The flowing medium was exchanged from the stationary phase medium to M9 medium 360 min after the stationary phase medium incubation started; the cell resumed its growth with no delay of time, assuring the preservation of its viability in the stationary phase medium. We confirmed that the rapid recovery of growth to the original level after the incubation in the stationary phase medium for almost all of the observed cells (14 cells out of the 15 observed cells).

There still remains the possibility that the growth stop in Figure 19(a) was caused by the deficiency of the essential nutrients in the medium. Thus we next supplemented the stationary phase medium with glucose and amino acids with the same amount that was added to prepare fresh M9 medium, and imposed this supplemented medium on exponentially growing cells (Figure 19(b)). The cell also stopped its growth and division in response to the supplemented medium even though it contained an enough amount of the nutrients; the growth stop was independent of the existence of the nutrients. Therefore, it is conceivable that the growth stop was caused by the diffusible communication signals in the stationary phase medium.

To characterize the speed of the response to the change of the flowing media, the transitions of growth rate every ten minutes around the time of the medium exchange from M9 medium to the stationary phase medium were examined (Figure 19(c)). The growth rate, *ν*, from time *t* min to (*t* + 10) min was defined as *ν* = (1/10) ln [*L*(*t* + 10)/*L*(*t*)] (min^−1^), where *L*(*t*) is cell length at the time of *t*, assuming that the length growth of *E. coli* occurs exponentially as, *L*(*t*) = *L*(*t_0_*) *e^ν(t−t0)^* (*t* > *t_0_*). Figure 19(c) shows the transition of the growth rate average of the four isolated cells in the four rooms of the observation area around the medium exchange. The growth rate between the 10 min and the 20 min since the medium exchange decreased to zero, showing that all the cells stopped their growth within 10 min in response to the signals in the stationary phase medium. This reveals the ability of *E. coli* to adapt its growth rate quickly to the diffusible communication signals.

To elaborate the growth suppression mechanism through the diffusible signals, we next diluted the nutrient-supplemented stationary phase medium with M9 medium at various concentrations and imposed it on exponentially growing cells in microchambers.

**Figure 20.**
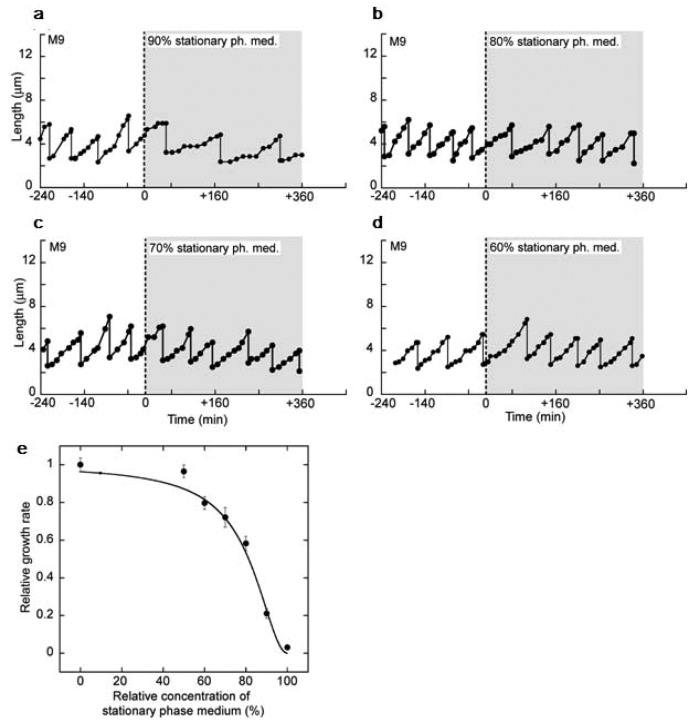
Single cell responses to diluted stationary phase media. (**a**) Single cell response of exponentially growing cell to 90% stationary phase medium. (**b**) Response to 80% stationary phase medium. (**c**) Response to 70% stationary phase medium. (**d**) Response to 60% stationary phase medium. (**e**) Relationship between growth rate and concentration of stationary phase medium. The error bars represent the standard errors (n = 14 for the 100%, and n = 4 for all the other points). The fitting curve represents: *ν_r_* = (1 − C)^2.0^/[0.038 + (1 − C)^2.0^].

Figure 20(a–d) show the responses of single isolated cells to the 90% (Figure 20(a)), the 80% (Figure 20(b)), the 70% (Figure 20(c)) and the 60% (Figure 20(d)) stationary phase media. The results show that the growth was suppressed instantly in response to the diluted media as was also seen in the responses to the 100% stationary phase medium. The extent of the suppression was weakened as the concentration of the stationary phase medium was lowered. Moreover, the cells in the diluted media maintained the suppressed-level growth rates without gradual decreases. These results suggest that cells quickly adjust their growth rate according to the present concentrations of the communication signals around them.

The relationship between growth rate and concentration of stationary phase medium is shown in Figure 20(e). The graph reveals that the growth suppression started from the 60% concentration of the medium and strengthened drastically. This non-linear relationship was fitted with a Hill equation: *ν_r_* = (1 − C)^2.0^/[0.038 + (1 − C)^2.0^], where*ν_r_* is a relative growth rate to that in M9 medium (the 0% stationary phase medium concentration) and *C* is a concentration of stationary phase medium.

The next question is whether the characteristics of a population are explainable from the single cell behaviors revealed above. To explore this, we measured the responses of batch culture cell populations to the stationary phase media. For this measurement, we inoculated *E. coli* cells into a 20-mL M9 medium. The cells were cultured at 37 °C by shake for 5 hours and make them reach the mid-exponential phase (optical density at 600 nm was *ca.* 0.1). The cultures were then centrifuged at 2,500 × g for 10 min, the supernatant being discarded thereafter. 20-ml nutrient-supplemented media at various stationary phase medium concentrations were added, and the cells in the precipitate were diffused by pipetting. The cells were again cultured at 37 °C by shake and the growths in the various initial concentrations of the stationary phase medium were measured.

**Figure 21.**
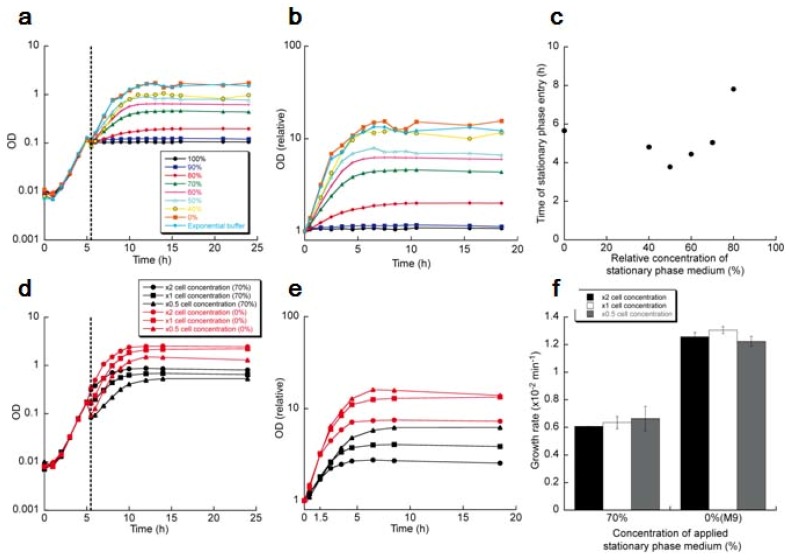
Response of cell populations. (**a**) Responses to stationary phase medium at various concentrations. The stationary phase media at the various concentrations were imposed on the cell populations in the mid-exponential phase; the growths in the imposed media were observed thereafter. The growth was measured by optical density at 600 nm. (**b**) Growth of populations after medium exchanges. The growths in the imposed media were enlarged with the initial cell concentrations normalized. (**c**) Time of stationary phase entry. The time of entering stationary phase (see text for the definition) was plotted against the concentration of the imposed stationary phase medium. (**d**) Effect of cell-to-cell contact. The cell populations in the mid-exponential phase were imposed to the 70% or the 0% stationary phase media at the ×2, ×1, or ×0.5 cell concentration relative to that before the medium exchanges. (**e**) Growths at different initial cell concentrations. The growths of the populations in (d) after the medium exchanges were enlarged with the initial cell concentrations normalized. (**f**) Growth rate at different initial cell concentrations. The growth rates of the populations in (d) for the first 1.5 hours were compared. The error bars represent the standard deviations (n = 3).

Figure 21(a) shows the growths of the cell populations measured by optical density at 600 nm. The relative growths after the medium exchange were enlarged in Figure 21(b), replotting with the initial cell concentrations normalized.

The growth of the population in the 100% stationary phase medium stopped instantly and stayed constant thereafter as was expected from the single cell observation in Figures 11–20. In the diluted supernatants, the populations took the suppressed-level growth rates in response to the medium exchanges, which is also expected from the single cell observation in Figure 20. However, an intriguing and unexpected characteristic was shown in the populations' growths: they entered stationary phases at the different cell concentrations without reaching the maximum cell concentration of the population whose medium was exchanged to the 0% stationary phase medium (M9 medium). There is no possibility that the difference in the final cell concentrations lies in the deficiency of the nutrients, for the diluted stationary phase medium was prepared by mixing M9 medium and the supplemented stationary phase medium; the concentration of the nutrients are the same as that of M9 medium in the case that the nutrients were completely deprived in the stationary phase medium, or more than that of M9 medium in the case that the nutrients were left in the stationary phase medium.

As another characteristic of the growth of the populations, the time to enter the stationary phases was compared between the populations (Figure 21(c)). The time to enter stationary phase medium was defined as the time for the logarithm of relative ODs in Figure 21(b) to reach to 0.95 log (Final OD). The result shows that the population whose medium was exchanged to the 80% stationary phase medium exhibited the significant delay to enter the stationary phase.

To examine the effect on growth of populations from cell-to-cell direct contact, we altered cell concentrations of batch cultures in the mid-exponential phase, exchanging the media from 20 mL M9 medium to 10 mL, 20 mL or 40 mL of the 70% stationary phase media or M9 medium, thereby compared the growths of the populations at the different initial cell concentrations where the cell-to-cell contact frequencies are different. Figure 21(d) is the growths of those cell populations. The relative growths after the medium exchanges were enlarged in Figure 21(e) with the initial cell concentration of each population normalized. The growth rates of the first 1.5 hours were measured in Figure 21(f), showing that the growth rates in the identical concentration of the stationary phase medium were the same despite the difference of the initial cell concentrations, that is, under the conditions where the cell-to-cell direct contact frequencies were different. This means that the cell-to-cell direct contact does not affect the growth rate; only the communication through diffusible signals affects the growth rates.

The result that cell-to-cell direct contact has no effect on growth promises to explain the characteristics of cell population from the results of the single cell observation that focused on the communication only through the diffusible signals. The results of the single cell observation are summarized as follows:

A cell determines its growth rate according to the concentration of the diffusible signals in the present immediate environment (Figures 19(a,b) and 20(a–d)). The relationship between growth rate and the concentration of the diffusible signals can be written as the Hill equation (Figure 20(e)).

In addition to the facts above, the information on how cells produce the signals is necessary to simulate population growth. Because this information was unobtainable from the single cell observation, we examined the two models, each of which postulates a specific simple signal production mechanism. In Model 1, we postulated that the signal production rate per unit cell volume was constant. On the other hand in Model 2, we postulated that the signal production rate per unit cell volume was proportional to the growth rate at that moment. With these postulates, we simulated the growths of populations at the various initial signal concentrations (data not shown). The both models reproduced the characteristic of the population growths in the experiment, that is, the entrance into the stationary phases without reaching the maximum cell density in the diluted stationary phase media. We also examined the time of entering the stationary phases, revealing that Model 2 reproduced the experimental result well that the population imposed the 80% stationary phase medium exhibited the significant delay to enter the stationary phase.

The result means that if we postulate the signal production rate per unit volume is proportional to the growth rate at that moment, the behaviors and characteristics of the cell population is explainable from the characteristics of single cells. Therefore, the macroscopic behavior of cell populations is understandable from the microscopic characteristics of individual cells that communicate and alter their growth states through the diffusible signals.

As shown above, we have demonstrated a new method of quantitatively measuring the sole effect of cell-to-cell communication through diffusible signals under the conditions where cell-to-cell direct contact is strictly avoided. Owing to the ability of this method to separate the two styles of cell-to-cell communication, the growth suppression observed in the single cell observation is clearly attributable to the communication through the diffusible signals in the stationary phase medium. In the conventional methods of measuring the effect of cell-to-cell communication such as observing cells on gel plates [28] and measuring the growths at high cell density in liquid media [67], the two styles of cell-to-cell interactions, through diffusible signals and through cell-to-cell contact, couldn't be strictly separated; the exact contribution of each style to the determination of individual cells' phenotypes and to the coordination of cellular behaviors were not measurable.

This method can be applied to measure the effect of cell-to-cell direct contact by enclosing a specific number of cells in one room of a microchamber and constantly flowing fresh medium; the cells in a room can communicate only through cell-to-cell direct contact in this condition. We indeed enclosed two cells in one room and measured their growths, detecting no suppression through cell-to-cell contact (data not shown). This is expectable from the result in Figure 21(d,e), in which we showed that the growth rates of the populations were not affected by cell-to-cell direct contact.

The single cell observation in this study shows that the diffusible signals in the stationary phase environment works instantly within 10 min on exponentially growing cells and that cells determine the growth rates according to the signal concentration. The results mean that the communication signals are one of the major determinants of individual cells' growth states, hence, of the growth phases of cell populations. Moreover, the fact that even exponentially growing cells responded instantly to the stationary phase medium suggests that cells make them always ready to respond to the signals even in an exponential phase.

The signals in the stationary phase medium have not been characterized yet. We have confirmed so far that the growth suppression effect was preserved in the incubation at 80 °C for 10 hours, and in the treatments of trypsin and carboxypeptidase Y. This suggests that the factors are neither protein nor peptide. It may be probable that several diffusible factors cooperatively work in the growth suppression.

The possibility that the growth suppression was caused by the low pH of the stationary phase medium (pH 5.9) compared with that of M9 medium (pH 6.8) can be denied because the growth rate in the low pH M9 medium (pH 5.8) was lower only by 20% than in the normal M9 medium. Although the low pH might contribute to the growth suppression to some extent, it cannot explain the instant and complete suppression of growth by the stationary phase supernatant.

### 3.8. Quantitative Measurement of Possible Damage Caused by 1064-nm Wavelength Optical Trapping of Escherichia coli Cells Using on-Chip Single Cell Cultivation System [77]

In the experiments above, we have used optical tweezers to control the numbers of cells within the microchambers. Thus, to distinguish whether the change of cells' property was caused by the change of environment or caused by the optical trapping, we need to quantify the possible damage to cells caused by the optical trapping of them. About those possible damage caused by optical trapping, Ashkin's group reported first that there is no damage to the growth and division of *E. coli*, with 1,064-nm, 80 mW optical trapping for several intervals of 10 min irradiations [45]. However, the following reports showed the potential damage caused by the optical trapping, e.g., damage on cells' propagatoin [78–80], the damage of cell's motility [81], and direct damage caused by the expression of stress response gene [82]. As these reports are qualitative report, we still cannot clarify how safe we can use the optical tweezers for cell handling.

Hence, we report here the results of quantitative optical trapping damage measurement in which we continuously followed the growth and division dynamics of isolated single cells of *E. coli* with comparing those of non-treated sister cells under a uniform and steady condition, in order to determine the magnitude of the effect between cells possessing identical genetic information and experience.

**Figure 22.**
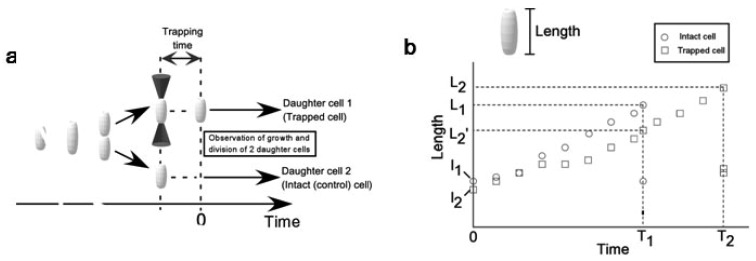
Procedure for damage measurement. (**a**) Direct comparison of two daughter cells. In our method, we directly compared two daughter cells derived from the same mother to measure the damage. At the division of a mother cell, we arbitrary chose either of the daughter cells and trapped it by optical tweezers. After trapping for pre-determined time, we stopped trapping it and compared the growth and division pattern between trapped and intact daughter cells. In this method, an intact daughter cell is used as a reference of the damage measurement. (**b**) Growth and division parameters. Simultaneous observation of two daughter cells gives time-course change of cell lengths of both cells. This graph holds the information of growth and division characteristics. We compared interdivision time and growth speed between trapped and intact daughter cells. See text for the definitions of interdivision time and growth speed.

**Figure 23.**
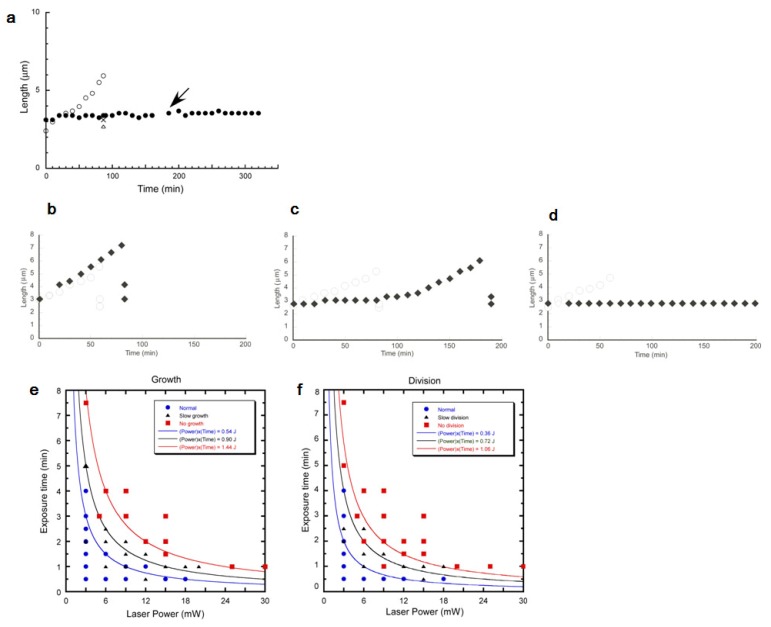
Damage to growth and division abilities at various laser power and trapping time conditions. (**a**) No growth and division in continuous trap. The graph shows time-course changes in lengths of both the continuously trapped daughter cell and the intact daughter cell. The length of trapped cell (filled circle points) showed almost no change in length in trapping (0–180 min) while the intact daughter cell grew and divided normally (open circle points). Though the trapped daughter cell was released from the trap at 180 min (black arrow), the length did not change for at least 140 min. even after the release. (**b**–**d**) Growth and division patterns of optically trapped cells under various trapping conditions. Three typical examples of growth and division patterns of trapped cells under various trapping conditions. (b) The trapped cell with no damage. It had almost the same growth speed and divided faster than the intact cell. (Laser power = 3 mW(Obj.), trapping time = 1 min). (c) The trapped cell with slower growth speed and longer interdivision time than the intact cell although it did grow and divide. (Laser power = 18 mW(Obj.), trapping time = 1 min). (d) The trapped cell with no growth and division. (Laser power = 15 mW(Obj.), trapping time = 2 min). (**e**) Damage estimated by their growth. (**f**) Damage estimated by their division. The blue circles in the graph represents the points of “normal”, the black triangles “slow”, and the red squares “no” growth, respectively.

In this experiment, we used the on-chip single cell cultivation system as described in Figure 6. In our protocol, one of two daughter cells was used as a target cell to which various trap conditions were applied. The other daughter cell was used as a reference. Figures 11–23(a) summarizes our protocol. At the beginning, we focused on a microchamber containing one cell in either of four rooms of a microchamber. When the cell divided into two daughter cells, one of them was arbitrary chosen, trapped by optical tweezers, and transported slowly to one of the other vacant rooms by moving a stage of a microscope. We released the cell after trapping for a pre-determined time. This release time was defined as time = 0. We kept recording the growth of both trapped and intact daughter cells simultaneously either until both of the two daughter cells divided or until the time reaches 180 minutes.

From the video data, we measured the time course change of cell lengths of two daughter cells (see Figure 22). The time-course data holds the information on the differences between two daughter cells in basic growth and division functions. As parameters, we defined division time (*T*) and growth speed (*v*). Division time is a time taken to divide from time = 0. Growth speed is defined as

(1) $$v=\frac{1}{T}\frac{L-l}{l}$$

where *L* is cell length just before the division and *l* is length at time = 0. We adopted these parameters to examine the damages on growth and division functions.

First, we examined whether an *E. coli* cell could grow and divide during continuous trap of optical tweezers. We trapped one fo two daughter cells with minimum force for optical trapping of cell, 3 mW power (at obj. lens position) continuously. As the rod shaped *E. coli* stands perpendicular to the light way of optical tweezers (*i.e.*, vertical; see Figure 22(a)), we cannot observe the shape of *E. coli* during its trapping. Thus, we released it for ten seconds from the trap every ten minutes and measured the length of the trapped cell. Figure 23(a) shows the time course of cell growth. The trapped cell (filled circles) didn't grow in a continuous trap for 180 min. On the other hand, the other intact daughter cell normally grew with exponential manner and divided in 88 min (open circles; initial lengths of divided two daughters, open triangle and cross “X”). After we finished 180 min laser irradiation (see arrow in Figure 23(a)), we continuously examined the change of the ability of the long-term trapped cells, and found no growth nor any division for at least 140 min. This result showed that *E. coli* cannot grow and divide both in and after the long-term trap of 3 hours even at the minimum laser power for optical trapping of cells, 3 mW (obj.). We found that a long-term trapping suppresses the growth and division abilities of *E. coli* regardless of its trapping power.

Next, we examined the damage from optical trapping under various trapping time and laser power conditions. For that, we changed trapping time from 0.5 min to 7.5 min and laser power from 3 mW to 30 mW. For the quantitative evaluation of the damage caused by the optical trapping, we compared the relative differences between the trapped and the intact daughter cells, and categorized into the three patterns as described above. As the growth speed and interdivision time of each individual cells are too variable to distinguish the regular growth speed and slower growth speed, the higher similarity of growth speed and interdivision time of sister cells was applied for comparing the results, *i.e.*, we used the intact sister cells as the standard growth curve to categorize the trapped sister cells. In the analysis, the difference in growth speed between sister cells (both free from the irradiation of laser trapping) was less than 4.0 × 10^−3^ min^−1^ with 25% CV.

Similarly, the difference in interdivision time between sister cells was less than 36 min with 25% CV. We therefore judged that the trapped cell was “Normal” when the differences between the trapped and intact daughter cells were within 25% difference. When the differences were larger than the 25% differences, even though they did grow or divide within 180min. we judged their growth or division was “Slow”. The conditions under which the trapped cell did not grow or divide at all within 180min were attributed to “No” growth and division conditions. Figure 23 also shows the typical examples of those three types. First, almost no difference between the trapped cell and the intact cell, which is usually observed in the cells trapped for a short time with weak laser power (Figure 23(b)). Second, slower growth and slower interdivision time, which is observed under the increased trapping time and laser power condition (Figure 23(c)). The third and finally, completely stopped growth and division case as described in Figure 23(d).

Figure 23(e,f) shows the damage dependences of laser power and trapping time in growth speed (Figure 23(e)) and in division time (Figure 23(f)). Each plot of the filled circles represents the one experimental result of those of the normal response of cell growth and division. Filled triangles are those of slower cells, and filled squares are those of no growth. The results showed that the damages on growth and division became more obvious when the cells were trapped with stronger laser power and for longer trapping time. Comparing Figure 23(e,f), we found that the damage intensity is larger on division time than on growth speed. There were several conditions under which the division was completely inhibited while growth speed was normal (for example, at 12 mW–1 min point).

Furthermore, the different type of damage (normal, slow, no growth) separated into three regions separated by the hyperbolic curves, *i.e.*, (laser power) × (trapping time) = constant. For example, when the total work was less than 0.54 J, growth tendency was normal, whereas the work larger than 1.44 J gives no growth tendency. Similarly, division tendency was normal if the total work was less than 0.36 J, and 1.06 J may stop the cell division activity. Those results of tendency suggest the damage depended on the total work applied to the cells from optical trapping, not caused by the non-linear factors. That is, the possible damage affected by the optical trapping might be estimated by the total energy applied to the cells.

Under the various trapping conditions of laser power and trapping time, we found the damage intensities on growth were larger than on division. Moreover, we found the damage could affect cell growth and division even under the minimum power of trapping even this trapping condition was a magnitude smaller than the previous reports [45,78–82].

The difference of the threshold intensities of damages on cell growth and division might mean that the optical trapping affected on the two different cell functions. The significance of this result for the application of optical handling to cell biology is that the trapping damage of cell division mechanism must be more sensitive than those of cell growth. For example, the maximum value of the safe “normal” trapping condition for growth was 18mJ, while that for division was 6 mJ. Thus we should apply optical handling for the study of bacterial division below 6 mJ, which is more tight condition than that of bacterial growth.

One possible candidate of the origin of those damages might be mutations in the genes coding proteins that act in DNA segregation or septation [83,84]. In our observations, however, the cells in the next generation produced from the damaged cells returned to the regular growth and division pattern (data not shown), indicating that the damage could not have been caused by mutations in these candidate genes. It is also known that the SOS or heat shock responses can lead to change their characteristics because they transiently inhibit the division mechanisms [84–86]. We must, however, stress the uniformity of the environmental condition in on-chip single cell cultivation system imposed on our cells: there is no reason to invoke these responses except for optical trapping. Moreover, since the slower elongation and division did not inherit to next generation, it is very improbable that this behavior was occasioned by any damage on gene itself. Hence we assert that the slowing and stopping of growth and division are occasioned by the intracellular leak of the reactions that temporarily inhibit the growth and division mechanism, which might be caused by the optical trapping prevents free movement of molecules for their reaction. Strengthening evidence for such stochastic intracellular reactions is provided by the observation that even in the regular growth and division pattern under uniform conditions, significantly large fluctuations in interdivision time (up to 33%) were observed [6].

Combining all our present results, optical trap completely suppresses both growth and division under continuous trap and after the long-term trap. And the damage linearly depends on the total energy (work) applied by optical trap and is more intense on division than on growth. It should be noted that these results could not have been obtained without the direct observation and competition of two sister cells under strict control of environmental conditions. This method of following the behavior of specific phenotypes of individual cells with strict control of their interactions should become a powerful tool in the near future for single cell based epigenetic studies, which are themselves rapidly acquiring importance as an essential element of post-genome research.

## 4. Conclusions

We have developed and used a series of new methods of understanding the meaning of genetic and epigenetic information in a life system exploiting microstructures fabricated on a chip. The most important contribution of this study was to be able to reconstruct the concept of a cell regulatory network from the “local” (molecules expressed at certain times and places) to the “global” (the cell as a viable, functioning system). Knowledge of epigenetic information, which we can control and change during cell lives, complements the genetic variety, and these two kinds are indispensable for living organisms. This new kind of knowlege has the potential to be the basis of cell-based biological and medical fields like those involving cell-based drug screening and the regeneration of organs from stem cells.
